# Almeric Paget Massage Corps

**Published:** 1915-11-20

**Authors:** 


### Almeric Paget Massage Corps.

At the request of Miss Essex French, the Hon. Secretary of this Corps, we have pleasure in publishing the following statement which she has sent :—

"May I be allowed, through the medium of your columns, to draw attention to the very pressing need for the treatment by massage and electricity for the wounded soldiers? The Almeric Paget Massage Corps has been in existence since the beginning of the war, and is the official organisation, recognised by the War Office, for the supply of masseurs and masseuses for the military hospitals and convalescent camps throughout the United Kingdom. The need for this form of treatment is increasing daily, and large camps are now being opened all over the country, besides those already in existence, where electrical and massage departments are to be a special feature. It is, therefore, necessary to procure the services of a very large number of masseuses in addition to those already working on the Corps, which now amount to about 700.

"The qualifications, without which no application for enrolment on the Almeric Paget Massage Corps can be entertained, are: (1) A certificate of the Incorporated Society of Trained Masseuses; or (2) a certificate of a Physical Training College recognised by the Ling Association; or (3) a certificate of one of the public hospital Schools of Massage; (4) a certificate, dated prior to January 1, 1916, of any of the Schools of Massage approved by the War Office, provided that the candidate can produce two satisfactory references from qualified doctors who can speak from personal knowledge of the candidate's work and character at any time during the three years immediately preceding the date of application for enrolment on the Corps. Applicants should forward their names, addresses, and qualifications as soon as possible to me at 39 Berkeley Square, London, W."

